# Long‐term effects of prenatal magnesium sulfate exposure on nervous system development in preterm‐born children

**DOI:** 10.1002/fsn3.3630

**Published:** 2023-09-10

**Authors:** Le Zhou, Xinghui Liu, Xiaoli Yan, Yingwei Liu, Yao Xie, Chuntang Sun

**Affiliations:** ^1^ Obstetrics and Gynecology Department, West China Second University Hospital Sichuan University Chengdu China; ^2^ Obstetrics and Gynecology Department The Southwest Hospital of the Army Medical University Chongqing China; ^3^ Obstetrics and Gynecology Department The First Affiliated Hospital of Chongqing Medical University Chongqing China; ^4^ Obstetrics and Gynecology Department Sichuan Academy of Medical Sciences – Sichuan Provincial People's Hospital Chengdu China

**Keywords:** gray matter volume, magnesium sulfate, preterm birth, structural magnetic resonance imaging, voxel‐based morphometry

## Abstract

This study used structural magnetic resonance imaging to analyze changes in the gray matter volume (GMV) of preterm‐born (PTB) and term‐born (TB) children to help elucidate the influence of magnesium sulfate treatment on the nervous system development. A total of 51 subjects were recruited, including 28 PTB and 23 TB children. The intelligence scale and MRI scan were completed at the corrected age of 10 to 16 years. A whole‐brain voxel‐wise analysis tested the main effect of the status (PTB without magnesium, PTB with magnesium, and TB) using a factorial design in SPM8. The mean volumes of the regions that showed significant group effects on the GMV after the FDR correction were extracted in the common space for each subject. Verbal and full‐scale intelligence quotient scores were significantly lower for PTB children without magnesium than for TB children; however, the scores of PTB children with magnesium and TB children were almost identical. Compared with TB children, PTB children had significantly reduced left straight gyrus and left inferior frontal gyrus GMVs; however, the volumes of PTB children with magnesium were closer to those of TB children. Changes in the GMV of the left inferior frontal gyrus were significantly correlated with full‐scale and verbal intelligence quotient scores, whereas the lower gestational age at the time of mgsou4 treatment led to a larger GMV of the left inferior frontal gyrus. Brain structural abnormalities could exist in PTB children. The GMVs of the left straight gyrus and left inferior frontal gyrus were significantly reduced in these children. The influence of magnesium sulfate treatment was not significant, but the cognitive levels of these children were significantly increased and almost identical to those of TB children. Initiation of magnesium sulfate treatment during gestation is negatively correlated with the left inferior frontal gyrus GMV.

## Graphical abstract

This study used structural magnetic resonance imaging to analyze changes in the gray matter volume of preterm‐born and term‐born children and aimed to provide imaging evidence to help elucidate the influences of magnesium sulfate treatment on nervous system development. We believe that our study makes a significant contribution to the literature because the protective effects of magnesium sulfate treatment before 32 weeks of gestation on fetal neurological development have been described by various studies, but only a few studies have investigated how magnesium sulfate treatment influences nervous system development. Further, we believe that this paper will be of interest to the readership of your journal because our results indicated that brain structural abnormalities could exist in preterm‐born children, even after adolescence. We found that gray matter volumes of the left straight gyrus and left inferior frontal gyrus were significantly reduced in these children. Furthermore, although the influence of magnesium sulfate treatment was not significant, the cognitive levels of these children were significantly increased and almost identical to those of term‐born children.

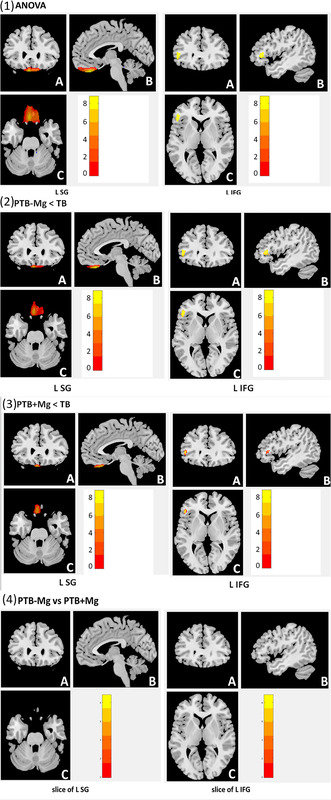

## 1 INTRODUCTION

The period from 3 months of gestation to 2 years after birth is a critical period for human brain development; furthermore, the third trimester of gestation is an especially crucial period for fetal brain development (Li et al., 2015). The third trimester of gestation mainly involves the proliferation of brain cells, the formation of the myelin sheath, and the development of enzyme systems that maintain brain functions (Basovich, 2010). Therefore, dissociation from the maternal system too early could influence intelligence development because of the reduction in fetal brain cell proliferation. Previous studies have demonstrated that preterm‐born (PTB) children have various issues, including problems with cognition, attention, behavior, and social adaptation (Lawn et al., 2014).

Recently, the value of prenatal magnesium sulfate (MgSO_4_) treatment for protecting the fetal nervous system has attracted increasing attention. In 1992, Kuban et al. (1992) first suggested that prenatal MgSO_4_ treatment could reduce the risk of intraventricular hemorrhage in PTB children, regardless of preeclampsia in their mothers. In 1995, Nelson and Grether (1995) performed a case‐control study and found that prenatal MgSO_4_ treatment led to significantly lower rates of cerebral palsy than those found in children born from mothers not treated with MgSO_4_. These findings were verified by subsequent studies that also demonstrated that prenatal MgSO_4_ treatment could reduce the rates of cerebral palsy, intraventricular hemorrhage, and periventricular leukomalacia, as well as the mortality rate of PTB children. However, the endpoints of all these studies were severe complications, such as cerebral palsy, and the exact changes in nervous system functions after MgSO_4_ treatment were not investigated.

Neuroimaging is a method of investigating subtle changes in the brain that could accurately evaluate cerebral structures and functions. Cerebral structural magnetic resonance imaging (MRI) is a noninvasive method of examining the physiological structures of the brain. Voxel‐based morphometry (VBM) is a structural MRI‐based cerebral morphology analysis method. In 2000, Ashburner and Friston (2000) first suggested that VBM allows for noninvasive investigation of cerebral morphology. VBM is a fully automatic data analysis method that could be used independently to detect changes in gray matter volume (GMV) and white matter volume through statistical analysis of whole‐brain data without any prior knowledge. Therefore, VBM results are objective and highly repeatable. Currently, the VBM technique is maturing gradually and has been applied to psychiatric diseases such as autism, post‐traumatic stress disorder, and social anxiety disorder. Several studies have also applied VBM to evaluate healthy populations, morphological changes of cerebral structures after some systemic training (such as learning a language or music), local cerebral morphological changes in different sexes, and changes induced by aging and development. This study aimed to use VBM to investigate the long‐term effects of MgSO_4_ on GM morphology in PTB children.

## 2 MATERIALS AND METHODS

### 2.1 Participants

A total of 51 subjects were recruited, including 28 PTB and 23 term‐born (TB) children. This study was registered with the Chinese Clinical Trial Registry, ChiCTR 2100050916. The guardians of the children were provided with a detailed information sheet about the study, and their written informed consent was obtained. The methods were performed in accordance with the approved guidelines.

The PTB children were delivered from 2004 to 2010. The intelligence scale and MRI scan were completed at the corrected age of 10–16 years. The MRI results of all subjects were first reviewed by a neuroradiologist to ensure that there were no structural abnormalities or flaws in the data quality. The inclusion criteria for the PB group were as follows: born at gestational ages ≥28 weeks and <32 weeks; Apgar score >7 in 5 min; transferred to the neonatal intensive care unit for treatment within 12 h after birth; normal results of cranial ultrasound or MRI; complete obstetrics and neonatal pediatrics clinical data available; right‐hand dominance; Chinese as the primary language; and good compliance.

The TB group was from another research project performed at the laboratory. The intelligence scale and MRI scan were completed before beginning this study. The control group included age‐matched, sex‐matched, handedness‐matched, and education‐matched children. The inclusion criteria for the TB group were as follows: born at gestational ages ≥37 and <42 weeks; birth weight >2500 and <4000 g; Apgar score >7 in 5 min; MgSO_4_ was not used during pregnancy; right‐hand dominance; Chinese as the primary language; and intelligence scale and MRI scan were completed between the ages of 10 and 16 years.

The exclusion criteria for all subjects were as follows: complications during pregnancy, such as pregnancy‐induced hypertension, heart disease, thyroid disease, hematological diseases, multiple gestation, chorioamnionitis, premature rupture of membranes, fetal growth restriction, fetal distress, and other diseases; long‐term medication use by the mother during pregnancy because of chronic disease; smoking, alcohol consumption, or illegal drug use by the mother during pregnancy; mental illness experienced by the mother during pregnancy; newborns with congenital malformations of important organs, genetic or metabolic diseases, chromosomal abnormalities, nervous system diseases, retinopathy of preterm infants, congenital or acquired severe infection, hypoglycemia, and other diseases or treatment including mechanical ventilation or blood transfusion in the neonatal intensive care unit; severe head trauma; serious diseases or disabilities of important organs and limbs; and abnormal brain structures found with MRI during the structural phase.

The intelligence quotient (IQ) of the subjects was measured using the Chinese Wechsler Intelligence Scale for Children (C‐WISC), administered by an experienced psychologist. The C‐WISC consists of two parts: the speech scale and the operation scale. The speech scale consists of six subtests: common sense, digit span, vocabulary, understanding, arithmetic, and similarity. The mapping and operation scale consists of five subtests: object patchwork, picture arrangement, building block patterns, and digital symbols. The scores of the 11 subtest scales can be combined with the speech scale, operation scale, and full‐scale scores. (Note: C‐WISC: National Collaborative Group on Revising the Wechsler Adult Intelligence Scale Revision of the Webster Adult Intelligence Scale).

### 2.2 Acquisition of imaging data

The whole‐brain T1‐weighted imaging dataset was acquired using a whole‐body 3.0‐T magnetic resonance system (EXCITE; General Electric, Milwaukee, WI, USA) with an eight‐channel phased‐array head coil. All participants were instructed to keep their eyes closed and to think of nothing in particular during acquisition. The sequence parameters were as follows: repetition time/echo time, 8.5 ms/3.4 ms; flip angle, 12°; field of view, 24 × 24 cm^2^; matrix, 256 × 256; slice thickness, 1 mm (no slice gap); and interslice gap, 0 mm. A total of 156 volumes were collected for each subject.

### 2.3 Image processing

The GMV was calculated using VBM after diffeomorphic anatomical registration through exponentiated lie algebra (DARTEL) (Ashburner & Friston, 2009) using Statistical Parametric Mapping software (SPM8; http://www.fil.ion.ucl.ac.uk/spm). DARTEL has been recommended instead of standard SPM normalization or the SPM‐unified segmentation approaches for whole‐brain and regional analyses without segmenting regions of interest (Ashburner & Friston, 2009).

The preprocessing of VBM‐DARTEL was performed in four steps. First, all original images were manually aligned on the anterior–posterior commissure line. Second, MR images were segmented into GM, white matter, and cerebrospinal fluid using the standard unified segmentation model in SPM8. Third, the DARTEL approach was applied for registration, normalization, and modulation, leaving the images in the DARTEL space (using this approach, a DARTEL template was created based on the deformation fields that were produced during the segmentation procedure, and all individual deformation fields were subsequently registered to this template). Normalization was achieved through nonlinear warping of the GM images to the DARTEL GM template in the Montreal Neurological Institute space, whereas modulation was used to ensure that the relative volumes of GM were preserved after the spatial normalization procedure. Fourth, the images were smoothed with an 8‐mm, full‐width, half‐maximum Gaussian kernel to correct nonlinear GMV for individual brain size for the statistical analysis. After spatial preprocessing, the smoothed, modulated, and normalized GM datasets were used for statistical analysis.

### 2.4 Statistical analysis

Demographic and clinical data comparisons were performed among groups with a one‐way analysis of variance (ANOVA), two‐sample *t*‐tests, and chi‐square tests using the Statistical Package for Social Sciences, version 23 (SPSS Inc.). Significance was set at *p* < .05.

GMV maps were analyzed in the context of the general linear model. A whole‐brain voxel‐wise analysis tested the main effect of the status (PTB without magnesium, PTB with magnesium, and TB) using a factorial design in SPM8. A voxel‐wise ANOVA was performed with the threshold set at *p* < .001, which was corrected by the false discovery rate (FDR) with the whole‐brain volume as a covariate of no interest. A contiguous cluster of at least 100 voxels was accepted as significant. Post hoc evaluations of significant ANOVA findings in these regions were then performed using secondary two‐tailed independent sample t‐tests. The GMV of the regions showing significant differences among the three groups was extracted using the Mars Bar toolbox.

The mean volumes of the regions that showed significant group effects on the GMV after the FDR correction were extracted in the common space for each subject. Partial correlations (two‐tailed) were also used to examine the relationship between the GMV and WISC scores (full‐scale IQ score, verbal IQ score, and performance IQ score) after controlling for age, sex, and gestational age at delivery. Multiple comparisons were also controlled using an FDR of *p* < .001.

## 3 RESULTS

### 3.1 Demographics

We enrolled 17 patients in the PTB with magnesium group, 11 patients in the PTB without magnesium group, and 23 patients in the TB group (only 23 healthy controls were successfully matched). The perinatal features and general characteristics of all eligible subjects are shown in Table 1. The sex of the subjects (*χ* ^2^ = 1.126; *p* = .570), age of the subjects (*F* = 0.050; *p* = .951), and mother's age at delivery (*F* = 1.671; *p* = .199) were not significantly different among the three groups. Furthermore, the gestational age (*p* = .492) and birth weight (*p* = .891) were not significantly different between the PTB with magnesium and PTB without magnesium groups.

**TABLE 1:** Demographics and clinical characteristics of the subjects.

Variables	PTB+Mg (*n* = 17)	PTB‐Mg (*n* = 11)	TB (*n* = 23)	*χ* ^2^或*F*	*p*
Gender (male/female)	11/6	5/6	12/11	1.126	.570^b^
Age (years)	14.05 ± 0.55	14.08 ± 0.52	14.11 ± 0.54	0.050	.951^a^
Gestational age (week)	30.87 ± 0.64^c^	30.69 ± 0.75^c^	39.53 ± 0.66	1069.994	.000^a^
Born weight (kg)	1413.24 ± 99.78^d^	1393.64 ± 98.77^d^	3120.22 ± 531.45	137.715	.000^a^
Mother's age at delivery (years)	28.00 ± 2.81	30.18 ± 4.02	29.13 ± 3.17	1.671	.199^a^
Full‐scale IQ	101.50 ± 9.86	94.22 ± 9.06^e^	103.57 ± 10.37^e^	3.338	.044^a^
Verbal IQ	104.71 ± 15.72	94.45 ± 12.64^f^	106.35 ± 15.4^f^	2.462	.096^a^
Performance IQ	98.29 ± 10.03	94.00 ± 11.52	100.78 ± 9.67	1.649	.203^a^

Abbreviations: PTB+Mg, preterm‐born children with magnesium sulfate; PTB‐Mg, preterm‐born children without magnesium sulfate; TB, term‐born children.

^a^ *p* value obtained by a two‐tailed two‐sample *t*‐test.

^b^ *p* value obtained by a two‐tailed Pearson chi‐square test.

^c^ The difference in maternal outcomes between PTB with magnesium and PTB without magnesium had statistical significance (*p* = .492).

^d^ The difference in maternal outcomes between PTB with magnesium and PTB without magnesium was statistical significance (*p* = 0.029).

^e^ The difference in maternal outcomes between PTB without magnesium and TB had statistical significance (*p* = .036).

^f^ The difference of maternal outcomes between PTB without magnesium and TB had statistical significance (*p* = .014).

The full‐scale IQ scores of subjects in the PTB without magnesium group were significantly lower than those of the TB group (*p* = .014). Additionally, the verbal IQ scores were also significantly lower in the PTB without magnesium group than in the TB group (*p* = .036). The performance IQ scores were not significantly different among the three groups (*p* = .203).

### 3.2 Effects of preterm birth on cerebral structures

**FIGURE 1:**
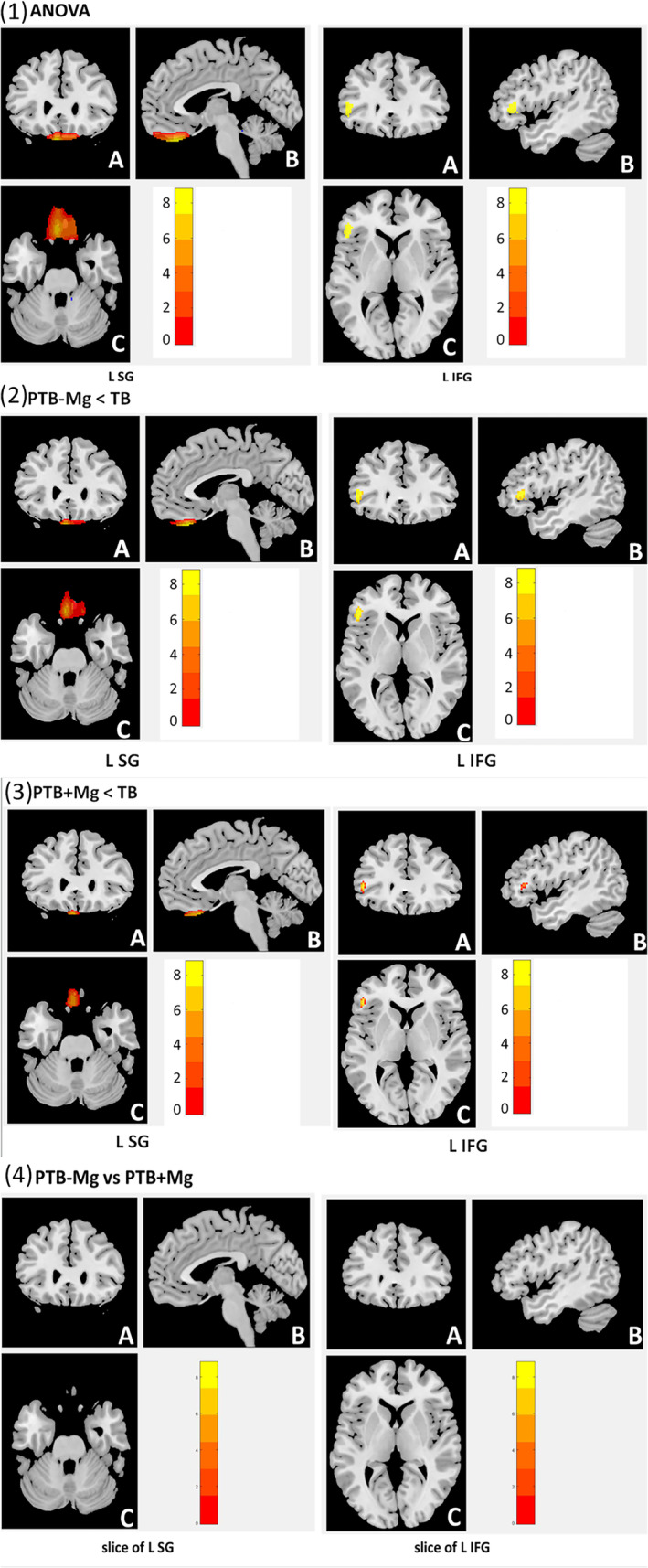
(1) Analysis of variance (ANOVA) comparison of the differences in the gray matter volume (GMV) of the three groups using the voxel‐based morphometry (VBM) method. (2) The results of comparing the GMV differences of the preterm‐born (PTB) without magnesium and term‐born (TB) groups using the VBM method. (3) The results of comparing the GMV differences of the PTB with magnesium and TB groups using the VBM method. (4) The results of comparing the GMV differences of the PTB with magnesium and PTB without magnesium groups using the VBM method.

A VBM analysis and one‐way ANOVA were conducted. Compared with the TB group, both the PTB with magnesium and PTB without magnesium groups had significantly lower GMV of the left straight gyrus and left inferior frontal gyrus (*p* < .05, FDR‐corrected). However, the difference between the PTB with magnesium group and the PTB without magnesium group was not statistically significant (*p* > .05, FDR‐corrected) (Table 2, Figure 1).

**TABLE 2:** Analysis of variance comparison of whole‐brain gray matter volumes among three groups.

Region	MNI coordinates			SPM	*F*/*t* value	*p* Value
	*X*	*Y*	*Z*			
ANOVA						
Left straight gyrus	−4	35	−31	580	147.197	.000
Left inferior frontal gyrus	−46	26	4	141	26.955	.000
PTB+Mg < TB						
Left straight gyrus	−4	32	−24	578		.000
Left inferior frontal gyrus	−47	22	12	125		.000
PTB‐Mg < TB						
Left straight gyrus	−3	40	024	486		.000
Left inferior frontal gyrus	−44	30	8	78		.000

Abbreviations: PTB+Mg, preterm‐born children with magnesium sulfate; PTB‐Mg, preterm‐born children without magnesium sulfate; TB, term‐born children.

### 3.3 Correlation between gray matter volume changes and clinical indicators

After age, gestational age, birth weight, and mother's age at delivery were adjusted, a partial correlation analysis of the C‐WISC scores of the three groups showed that the GMV of the left inferior frontal gyrus was significantly correlated with the full‐scale IQ and verbal IQ scores (*p* < .05) Table 3.

**TABLE 3:** Partial correlation analysis of the changes in gray matter volume and clinical data.

	Left straight gyrus		Left inferior frontal gyrus	
	*r*	*p*	*r*	*p*
Full‐scale IQ	−0.122	.419	0.386	.027
Verbal IQ	−0.175	.246	0.294	.034
Performance IQ	0.014	.926	0.033	.829

### 3.4 Observations of the preterm born with magnesium group

Seventeen subjects were included in the PTB with magnesium group during this study, and their MgSO_4_ treatment was performed using a loading dose of 4.0 g and intravenous drip within 30 min that was gradually reduced to 1 g/h for maintenance. The MgSO_4_ treatment for all subjects was performed before 32 weeks of gestation and lasted for 1–6 days. The mean blood magnesium concentration was 1.5–2.5 mmol/L. However, the blood magnesium concentration of three pregnant women was not measured. The general characteristics and drug therapy processes of the subjects in the PTB with magnesium group are shown in Table S1.

**FIGURE 2:**
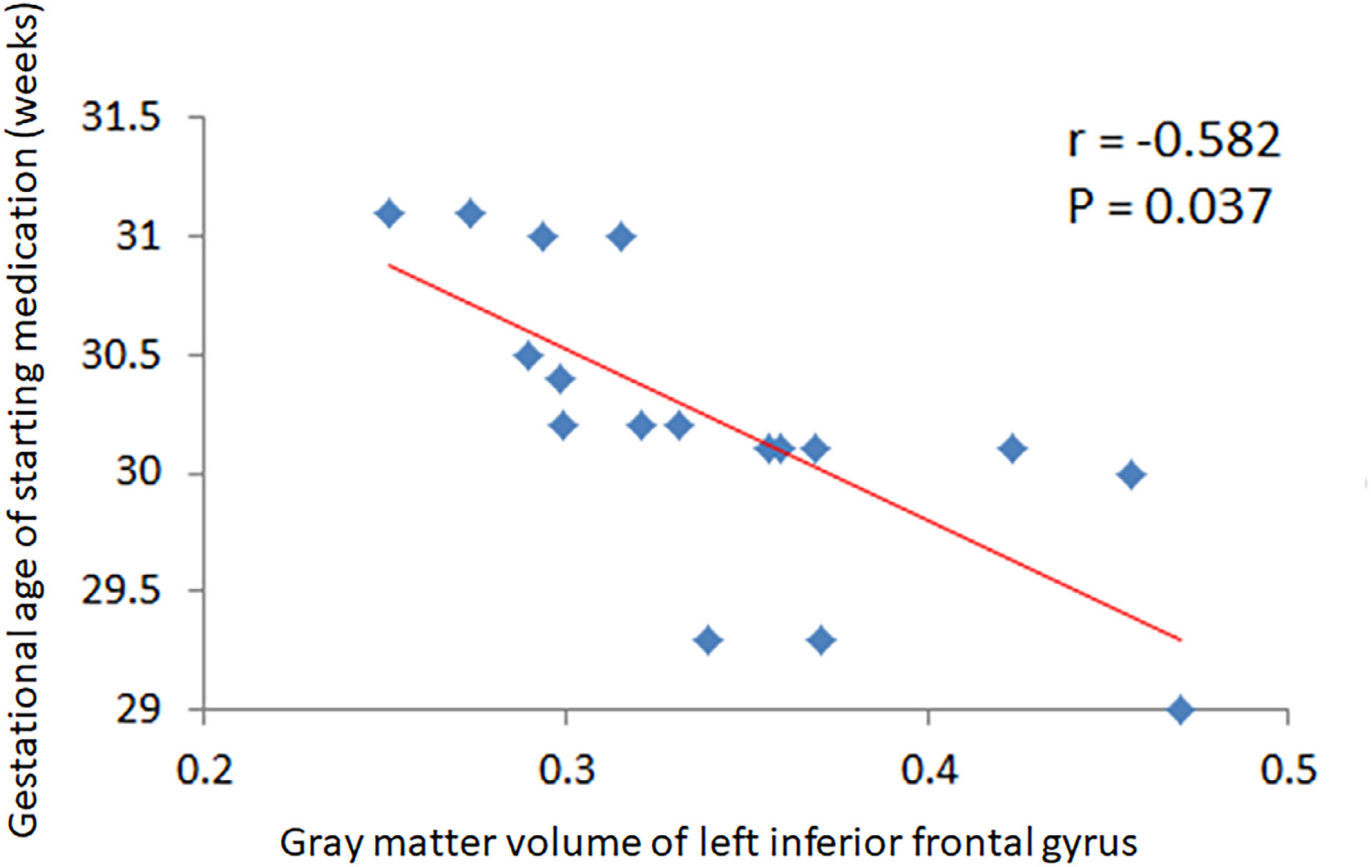
Scatter plot of the correlation analysis of the gray matter volume of the left inferior frontal gyrus and the gestational week when magnesium sulfate was administered.

After the possible confounding factors, such as sex, age, gestational age, birth weight, and mother's age at the time of delivery, were adjusted, a partial correlation analysis was performed to explore the correlations of gestational age at the time of MgSO_4_ treatment, treatment duration, and blood concentration of magnesium in cerebral regions with GMV changes. The results showed that the gestational age at the time of MgSO_4_ treatment was negatively correlated with the GMV of the left inferior frontal gyrus (*r* = −0.582; *p* = .037) (Table 4 and Figure 2). The lower the gestational age at the time of mgsou4 treatment, the larger the GMV of the left inferior frontal gyrus. However, the treatment duration and blood concentration of magnesium were not significantly correlated with GMV changes (*p* > .05).

**TABLE 4:** Partial correlation analysis of clinical indicators for magnesium sulfate and gray matter volume changes in brain regions.

	Left straight gyrus		Left inferior frontal gyrus	
	*r*	*p*	*r*	*p*
Gestational week of starting medication (week)	0.064	.844	**−0.582**	**.037**
Medication duration (days)	−0.123	.703	0.107	.740
Mean blood magnesium concentration (mmol/L)	−0.477	.232	0.408	.316

## 4 DISCUSSION

This study compared the whole‐brain GMV of PTB and TB children 10–16 years of age. The results demonstrated that the PTB group had cerebral structural abnormalities during childhood and that the cognitive levels of subjects who received MgSO_4_ treatment were significantly higher. The gestational age at the time of starting MgSO_4_ treatment was correlated with the GMV of the inferior frontal gyrus.

The third trimester is the “golden period” for fetal brain development (Hüppi et al., 1998). The fetal brain weight at 34 weeks of gestation is approximately only 65% that of TB newborns, and the gyri and sulcus are also incomplete (Ball et al., 2014). Fetal brain development is accelerated during the third trimester, when neurons, synapses, axons, and the myelin sheath are extremely active (Kostović & Judas, 2010; Tau & Peterson, 2010). Approximately 40,000 new synapses per second form between 32 and 37 weeks of gestation, and synaptic metaplasticity is highly evident (Tau & Peterson, 2010). During this period, the inhibitory γ‐aminobutyric acid neurons that have important roles in cerebral cortical output undergo sudden and accelerated proliferation (Xu et al., 2011). Therefore, the third trimester is critical for fetal nervous system development (Partridge et al., 2004). Dissociation from the maternal system too early could lead to a substantial reduction of fetal cerebral cells. Additionally, the germinal matrix and self‐regulation of cerebral blood vessels are not completely mature in PTB newborns; therefore, the sensitivity of cerebral tissues to ischemia and hypoxia is elevated. As a result, acute ischemia and hypoxia could easily induce injuries in regions with an active metabolism, such as the basal ganglia (Nagy et al., 2003). Because of the insufficient secretion of adrenocortical hormones, insulin, and thyroid hormones that could promote cerebral tissue development, the maturity of the brain in PTB newborns is significantly less than that in TB newborns (Walch et al., 2009).

This study showed that cerebral structural abnormalities still exist in PTB children during childhood and that the GMV of the left straight gyrus and left inferior frontal gyrus were significantly reduced. The position of the inferior frontal gyrus is lowest in the prefrontal gyrus, and the prefrontal lobe is an important node of various brain networks (Bonelli & Cummings, 2007). A study performed by Petersen and Posner (2012) suggested that the inferior frontal gyrus is a component of the ventral attention system, which has the capability of bottom‐up reorientation. More specifically, this system is associated with the active tracking of targets and has been considered part of the network responding to sensory stimulation. The straight gyrus is located at the bottom of the frontal lobe and is part of the longitudinal division closely adjacent to the inferior frontal gyrus, which is connected to the medial superior frontal gyrus and extends from the anterior cingulated gyrus to the prefrontal lobe (Roiz‐Santiáñez et al., 2012). Abnormal GM structures could lead to consequent influences on distal and adjacent cerebral regions and prevent these regions from receiving input from the damaged cortex (Jackson et al., 2017). Both the inferior frontal gyrus and straight gyrus are in the frontal lobe, which is closely associated with cognition, emotion, language, and memory (Bonelli & Cummings, 2007). Menegaux et al. (Narberhaus et al., 2009) found that even at age 20 years, the volume of the bilateral inferior frontal gyri, which manifested as a relatively immature frontal lobe, was still lower in PTB subjects than in TB subjects. Isaacs et al. (2004) compared the IQ scores of children 7.5–8 years old with normal neurological functions who were born at ≤30 weeks of gestation with those of their age‐matched TB counterparts and found that the reduction in IQ scores was associated with the decreased GMV of the parietal lobe and temporal lobe.

The findings of this study demonstrated that the IQ scores and speech capabilities of PTB children were still lower than those of TB children during childhood. During pregnancy, fetuses are exposed to an environment with rhythmic sound, which includes low‐frequency background sounds in utero (maternal cardiac rhythm and body noise) and familiar pronunciations and tones (such as the mother's voice). If the fetus is dissociated from the uterus too early, then the lack of rhythm of the environmental sound stimuli could influence language development (Herold et al., 2008), and the earlier the gestational age, the more delayed the language development (Schirmer et al., 2006). A prospective study performed by Allin et al. (2008) involving 94 PTB children showed that when they were 15–19 years old, the mental development as well as verbal and performance IQ scores of the PTB group were significantly lower than those of the TB group. A paired t‐test showed that the language fluency of the TB group increased significantly during adolescence, but it did not increase significantly in the PTB group. A study performed by Tanskanen et al. (2011) investigated PTB (average gestational age at birth, 34.6 weeks; average body weight at birth, 2159 g) and TB subjects at the ages of 33–35 years and found that academic achievement and educational level during adulthood were significantly lower and language learning performance was significantly poorer in the PTB group than in the TB group. PTB children are at higher risk for attention deficiency and hyperactivity disorder, which mainly manifest as learning difficulties or borderline intelligence levels in children. Attention deficiency in PTB children generally appears secondary to behavioral issues and has long‐term influences on subsequent cognitive development and social adaptability (Gascoin & Flamant, 2013).

A further correlation analysis during this study showed that the full‐scale IQ and verbal IQ C‐WISC scores were correlated with GMV changes in the inferior frontal gyrus. The superior frontal gyrus participates in the regulation of the emotional process, advanced cognition, language, and social adaptability, and changes in the superior frontal gyrus could induce cognitive impairment and delayed language development (Bonelli & Cummings, 2007). Nosarti et al. (2007) performed a study of 218 PTB children (gestational age <33 weeks) and 128 TB children when they were 14–15 years old and found that the GMV of the frontal lobe was significantly lower in the PTB group than in the TB group; furthermore, the verbal and performance IQ scores were significantly lower in the PTB group. These findings demonstrated that preterm birth could influence and delay the development and maturation of neurons in these cerebral regions, consequently delaying language development and the motor nerve network. Therefore, cognitive disorders, low reading comprehension capabilities, and lower movement capabilities could occur in PTB children.

All PTB subjects included in this study were born at 28–32 weeks of gestation and did not have severe preterm‐related complications or neuropsychiatric disorders. The findings of this study demonstrated that MgSO_4_ treatment did not substantially influence the cerebral structures; however, the cognitive level of PTB children treated with MgSO_4_ was significantly higher than that of PTB children not treated with MgSO_4_. The cognitive level of PTB children treated with MgSO_4_ was comparable to that of TB children, which could be attributable to changes in cerebral functions.

Finally, this study found that GMV changes in the inferior frontal gyrus were correlated with the gestational week when MgSO_4_ treatment was started; the earlier the treatment was started, the larger the inferior frontal gyrus GMV. Various guidelines have described that MgSO_4_ treatment before 32 weeks of gestation has protective effects on fetal nervous system functions (American College of Obstetricians and Gynecologists’ Committee on Practice Bulletins—Obstetrics, 2016; Preterm Labour and Birth, 2015). The World Health Organization (WHO Recommendations on Interventions to Improve Preterm Birth Outcomes, 2015) recommended that MgSO_4_ be routinely used to protect the nervous system of children born before 32 weeks of gestation (single or multiple gestations); the earlier the gestation week when treatment is started, the more significant the treatment effects. The third trimester is an important period for nervous system development. The occurrence of PTB could possibly change the trajectory of nervous system development, whereas influences on cerebral regions are more considerable when development is more delayed (Carmichael & Price, 1995). MgSO_4_ is a neuroprotective agent, and the more delayed cerebral regions could be more protected by MgSO_4_. However, for all the PTB children included in this study who received MgSO_4_, the treatment was started at 29–32 weeks of gestation.

## 5 CONCLUSION AND LIMITATIONS

The VBM method was used to analyze the whole‐brain GMV in PTB and TB children 10–16 years of age. The findings demonstrated that cerebral structural abnormalities exist in PTB children, even during childhood, and that the GMV is significantly reduced in the left straight gyrus and left inferior frontal gyrus. The influence of MgSO_4_ treatment on cerebral structures is not significant; however, the cognitive level of the subjects increased substantially. This needs to be investigated by further studies of the cerebral function network. Starting MgSO_4_ treatment at an earlier gestational age is correlated with closer to normal GMV in the left inferior frontal gyrus. However, the number of subjects included in this study was relatively small, and more studies are needed to further investigate. And the included subjects were born in different hospitals. Their clinical data are incomplete, which prevents us from conducting further research.

## AUTHOR CONTRIBUTIONS

CT Sun developed the study design, provided methodological advice, and supervised the conduct of the study. L Zhou, XL Yan, YW Liu and Y Xie collected the data. L Zhou and XL Yan He performed the data analysis. YW Liu and Y Xie generated the figures and tables. L Zhou drafted the manuscript. All authors reviewed and approved the final tex.

## FUNDING INFORMATION

This study was supported by the National Key Research and Development Program “Reproductive Health and Prevention and Control of Major Birth Defects” (No. 2016YFC1000400) and “Health Commission of Sichuan Province” (No. 17ZD007) of China.

## CONFLICT OF INTEREST STATEMENT

The authors report no conflicts of interest.

## ETHICAL APPROVAL

The application of this study was approved by the Ethics Committee of the West China Second University Hospital of Sichuan University (approval number: 2021173) and registered with the Chinese Clinical Trial Registry, ChiCTR 2,100,050,916.

## Supporting information


Table S1

## Data Availability

The data that support the findings of this study are available from the corresponding author upon reasonable request.

## References

[fsn33630-bib-0001] Allin, M. , Walshe, M. , Fern, A. , Nosarti, C. , Cuddy, M. , Rifkin, L. , Murray, R. , Rushe, T. , & Wyatt, J. (2008). Cognitive maturation in preterm and term born adolescents. Journal of Neurology, Neurosurgery, and Psychiatry, 79, 381–386. 17682017 10.1136/jnnp.2006.110858

[fsn33630-bib-0002] American College of Obstetricians and Gynecologists’ Committee on Practice Bulletins—Obstetrics . (2016). Practice Bulletin No. 171: Management of Preterm Labor. Obstetrics and Gynecology, 128, e155–e164. 27661654 10.1097/AOG.0000000000001711

[fsn33630-bib-0003] Ashburner, J. , & Friston, K. J. (2000). Voxel‐based morphometry—The methods. NeuroImage, 11, 805–821. 10860804 10.1006/nimg.2000.0582

[fsn33630-bib-0004] Ashburner, J. , & Friston, K. J. (2009). Computing average shaped tissue probability templates. NeuroImage, 45, 333–341. 19146961 10.1016/j.neuroimage.2008.12.008

[fsn33630-bib-0005] Ball, G. , Aljabar, P. , Zebari, S. , Tusor, N. , Arichi, T. , Merchant, N. , Robinson, E. C. , Ogundipe, E. , Rueckert, D. , Edwards, A. D. , & Counsell, S. J. (2014). Rich‐club organization of the newborn human brain. Proceedings of the National Academy of Sciences of the United States of America, 111, 7456–7461. 24799693 10.1073/pnas.1324118111 PMC4034228

[fsn33630-bib-0006] Basovich, S. N. (2010). The role of hypoxia in mental development and in the treatment of mental disorders: A review. Bioscience Trends, 4, 288–296. 21248426

[fsn33630-bib-0007] Bonelli, R. M. , & Cummings, J. L. (2007). Frontal‐subcortical circuitry and behavior. Dialogues in Clinical Neuroscience, 9, 141–151. 17726913 10.31887/DCNS.2007.9.2/rbonelli PMC3181854

[fsn33630-bib-0008] Carmichael, S. T. , & Price, J. L. (1995). Limbic connections of the orbital and medial prefrontal cortex in macaque monkeys. The Journal of Comparative Neurology, 363, 615–641. 8847421 10.1002/cne.903630408

[fsn33630-bib-0009] Gascoin, G. , & Flamant, C. (2013). Long‐term outcome in context of intra uterine growth restriction and/or small for gestational age newborns. Journal de Gynecologie, Obstetrique et Biologie de la Reproduction, 42, 911–920. 24220276 10.1016/j.jgyn.2013.09.014

[fsn33630-bib-0010] Herold, B. , Höhle, B. , Walch, E. , Weber, T. , & Obladen, M. (2008). Impaired word stress pattern discrimination in very‐low‐birthweight infants during the first 6 months of life. Developmental Medicine and Child Neurology, 50, 678–683. 18754917 10.1111/j.1469-8749.2008.03055.x

[fsn33630-bib-0011] Hüppi, P. S. , Warfield, S. , Kikinis, R. , Barnes, P. D. , Zientara, G. P. , Jolesz, F. A. , Tsuji, M. K. , & Volpe, J. J. (1998). Quantitative magnetic resonance imaging of brain development in premature and mature newborns. Annals of Neurology, 43, 224–235. 9485064 10.1002/ana.410430213

[fsn33630-bib-0012] Isaacs, E. B. , Edmonds, C. J. , Chong, W. K. , Lucas, A. , Morley, R. , & Gadian, D. G. (2004). Brain morphometry and IQ measurements in preterm children. Brain, 127, 2595–2607. 15371289 10.1093/brain/awh300

[fsn33630-bib-0013] Jackson, R. L. , Bajada, C. J. , Rice, G. E. , Cloutman, L. L. , & Lambon Ralph, M. A. (2017). An emergent functional parcellation of the temporal cortex. NeuroImage, 155, 503–512. 28411156 10.1016/j.neuroimage.2017.04.016 PMC5518769

[fsn33630-bib-0014] Kostović, I. , & Judas, M. (2010). The development of the subplate and thalamocortical connections in the human foetal brain. Acta Paediatrica, 99, 1119–1127. 20367617 10.1111/j.1651-2227.2010.01811.x

[fsn33630-bib-0015] Kuban, K. C. , Leviton, A. , Pagano, M. , Fenton, T. , Strassfeld, R. , & Wolff, M. (1992). Maternal toxemia is associated with reduced incidence of germinal matrix hemorrhage in premature babies. Journal of Child Neurology, 7, 70–76. 1552156 10.1177/088307389200700113

[fsn33630-bib-0016] Lawn, J. E. , Blencowe, H. , Oza, S. , You, D. , Lee, A. C. , Waiswa, P. , Lalli, M. , Bhutta, Z. , Barros, A. J. , Christian, P. , Mathers, C. , Cousens, S. N. , & Lancet Every Newborn Study Group . (2014). Every newborn: Progress, priorities, and potential beyond survival. Lancet, 384, 189–205. 24853593 10.1016/S0140-6736(14)60496-7

[fsn33630-bib-0017] Li, K. , Sun, Z. , Han, Y. , Gao, L. , Yuan, L. , & Zeng, D. (2015). Fractional anisotropy alterations in individuals born preterm: A diffusion tensor imaging meta‐analysis. Developmental Medicine and Child Neurology, 57, 328–338. 25358534 10.1111/dmcn.12618

[fsn33630-bib-0018] Nagy, Z. , Westerberg, H. , Skare, S. , Andersson, J. L. , Lilja, A. , Flodmark, O. , Fernell, E. , Holmberg, K. , Böhm, B. , Forssberg, H. , Lagercrantz, H. , & Klingberg, T. (2003). Preterm children have disturbances of white matter at 11 years of age as shown by diffusion tensor imaging. Pediatric Research, 54, 672–679. 12904607 10.1203/01.PDR.0000084083.71422.16

[fsn33630-bib-0019] Narberhaus, A. , Lawrence, E. , Allin, M. P. , Walshe, M. , McGuire, P. , Rifkin, L. , Murray, R. , & Nosarti, C. (2009). Neural substrates of visual paired associates in young adults with a history of very preterm birth: Alterations in fronto‐parieto‐occipital networks and caudate nucleus. NeuroImage, 47, 1884–1893. 19376244 10.1016/j.neuroimage.2009.04.036

[fsn33630-bib-0020] Nelson, K. B. , & Grether, J. K. (1995). Can magnesium sulfate reduce the risk of cerebral palsy in very low birthweight infants. Pediatrics, 95, 263–269. 7838646

[fsn33630-bib-0021] Nosarti, C. , Giouroukou, E. , Micali, N. , Rifkin, L. , Morris, R. G. , & Murray, R. M. (2007). Impaired executive functioning in young adults born very preterm. Journal of the International Neuropsychological Society, 13, 571–581. 17521479 10.1017/S1355617707070725

[fsn33630-bib-0022] Partridge, S. C. , Mukherjee, P. , Henry, R. G. , Miller, S. P. , Berman, J. I. , Jin, H. , Lu, Y. , Glenn, O. A. , Ferriero, D. M. , Barkovich, A. J. , & Vigneron, D. B. (2004). Diffusion tensor imaging: Serial quantitation of white matter tract maturity in premature newborns. NeuroImage, 22, 1302–1314. 15219602 10.1016/j.neuroimage.2004.02.038

[fsn33630-bib-0023] Petersen, S. E. , & Posner, M. I. (2012). The attention system of the human brain: 20 years after. Annual Review of Neuroscience, 35, 73–89. 10.1146/annurev-neuro-062111-150525 PMC3413263 22524787

[fsn33630-bib-0024] Preterm Labour and Birth . (2015). National Institute for Health and Care Excellence (UK). 26632624

[fsn33630-bib-0025] Roiz‐Santiáñez, R. , Pérez‐Iglesias, R. , Ortíz‐García de la Foz, V. , Tordesillas‐Gutiérrez, D. , Mata, I. , González‐Mandly, A. , Pazos, A. , Tabarés‐Seisdedos, R. , Vázquez‐Barquero, J. L. , & Crespo‐Facorro, B. (2012). One year longitudinal study of the straight gyrus morphometry in first‐episode schizophrenia‐spectrum patients. Psychiatry Research, 202, 80–83. 22595509 10.1016/j.pscychresns.2011.10.001

[fsn33630-bib-0026] Schirmer, C. R. , Portuguez, M. W. , & Nunes, M. L. (2006). Clinical assessment of language development in children at age 3 years that were born preterm. Arquivos de Neuro‐Psiquiatria, 64, 926–931. 17220997 10.1590/s0004-282x2006000600007

[fsn33630-bib-0027] Tanskanen, P. , Valkama, M. , Haapea, M. , Barnes, A. , Ridler, K. , Miettunen, J. , Murray, G. K. , Veijola, J. M. , Jones, P. B. , Taanila, A. M. , & Isohanni, M. K. (2011). Is prematurity associated with adult cognitive outcome and brain structure. Pediatric Neurology, 44, 12–20. 21147382 10.1016/j.pediatrneurol.2010.07.002

[fsn33630-bib-0028] Tau, G. Z. , & Peterson, B. S. (2010). Normal development of brain circuits. Neuropsychopharmacology, 35, 147–168. 19794405 10.1038/npp.2009.115 PMC3055433

[fsn33630-bib-0029] Walch, E. , Chaudhary, T. , Herold, B. , & Obladen, M. (2009). Parental bilingualism is associated with slower cognitive development in very low birth weight infants. Early Human Development, 85, 449–454. 19356865 10.1016/j.earlhumdev.2009.03.002

[fsn33630-bib-0030] . (2015). WHO recommendations on interventions to improve preterm birth outcomes. World Health Organization. 26447264

[fsn33630-bib-0031] Xu, G. , Broadbelt, K. G. , Haynes, R. L. , Folkerth, R. D. , Borenstein, N. S. , Belliveau, R. A. , Trachtenberg, F. L. , Volpe, J. J. , & Kinney, H. C. (2011). Late development of the GABAergic system in the human cerebral cortex and white matter. Journal of Neuropathology and Experimental Neurology, 70, 841–858. 21937910 10.1097/NEN.0b013e31822f471c PMC3193835

