# Supplementary material for: Long‐term effects of prenatal magnesium sulfate exposure on nervous system development in preterm‐born children
Source: Food Sci Nutr. 2023 Sep 10;11(11):7061–9. doi: 10.1002/fsn3.3630 (PMC10630835; doi:10.1002/fsn3.3630)
Supplement: Supplementary file 1 — Table S1 [file FSN3-11-7061-s001.docx]

**Supplementary Table 1. Clinical data of the preterm delivery with magnesium sulphate treatment group**

| N | sex | Gestational age (age) | Birth weight (g) | Subjects’ age (year) | Age of mother's delivery (year) | Gestational week of starting medication（week） | Medication duration (days) | Mean blood magnesium concentration（mmol/L） |
| --- | --- | --- | --- | --- | --- | --- | --- | --- |
| 1 | F | 31.5 | 1525 | 17.42 | 29 | 31 | 4 | 1.75 |
| 2 | F | 30 | 1275 | 17.75 | 28 | 29.3 | 2 | 1.8 |
| 3 | M | 30.3 | 1250 | 16.33 | 30 | 30.2 | 1 | - |
| 4 | M | 31.2 | 1430 | 17.83 | 31 | 30.2 | 4 | 1.3 |
| 5 | M | 31.3 | 1505 | 16.75 | 29 | 31.1 | 2 | 1.8 |
| 6 | F | 31.4 | 1560 | 17.92 | 27 | 30.1 | 2 | 2 |
| 7 | M | 30.1 | 1410 | 17.16 | 27 | 30 | 1 | - |
| 8 | M | 30.5 | 1320 | 16.5 | 27 | 30.1 | 3 | 1.9 |
| 9 | F | 29.5 | 1280 | 16.66 | 26 | 29 | 3 | 2.15 |
| 10 | M | 30.4 | 1330 | 17.58 | 30 | 29.3 | 6 | 1.55 |
| 11 | F | 31.4 | 1335 | 17.08 | 27 | 30.5 | 5 | 2.1 |
| 12 | M | 31.2 | 1490 | 16.16 | 27 | 30.2 | 2 | 2.4 |
| 13 | M | 31 | 1455 | 17.42 | 33 | 30.4 | 1 | - |
| 14 | F | 31.3 | 1465 | 17.33 | 30 | 31 | 2 | 2.5 |
| 15 | M | 30.6 | 1375 | 16.42 | 21 | 30.1 | 4 | 2.2 |
| 16 | F | 31.6 | 1490 | 16.83 | 30 | 31.1 | 4 | 2 |
| 17 | M | 31.5 | 1530 | 16.83 | 24 | 30.1 | 5 | 2.1 |
